# Vaginal resistome redundancy is associated with microbial metabolic potential and host glycomic features in endometriosis

**DOI:** 10.1038/s41522-026-01100-w

**Published:** 2026-07-23

**Authors:** Zhe Pan, Louise Glover, Fiona Reidy, Fionnuala M. McAuliffe, Mary Wingfield, Douwe van Sinderen, Radka Saldova, John MacSharry

**Affiliations:** 1 APC Microbiome Ireland, University College Cork, Cork, Ireland; 2 School of Microbiology, University College Cork, Cork, Ireland; 3 Merrion Fertility Clinic and National Maternity Hospital, Dublin, Ireland; 4 UCD Perinatal Research Centre, University College Dublin, National Maternity Hospital, Dublin, Ireland; 5 National Institute for Bioprocessing Research and Training (NIBRT), Belfield, Blackrock, Co, Dublin, Ireland; 6 UCD School of Medicine, College of Health and Agricultural Science (CHAS), University College Dublin, Dublin, Ireland; 7 CÚRAM, Science Foundation Ireland Research Centre for Medical Devices, Biomedical Sciences, University of Galway, Galway, Ireland

**Keywords:** Computational biology and bioinformatics, Microbiology

## Abstract

The vaginal microbiome plays a critical role in reproductive health, yet most studies remain descriptive and functional features connecting microbial communities to host physiology remain poorly understood. In endometriosis, alterations in the vaginal microbiome and host glycosylation have been reported, yet ecological properties connecting these systems remain unclear. We investigated whether vaginal resistome functional redundancy (FR) represents a functional link between microbial community structure, microbial metabolic capacity, and host glycomic variation across endometriosis severity. Shotgun metagenomic sequencing was performed on high vaginal swabs from women with healthy controls, minimal/mild, and moderate/severe endometriosis (n = 40). While total antibiotic resistance genes (ARG) abundance remained relatively stable across disease groups, resistome FR increased significantly in moderate/severe endometriosis, reflecting a transition from low-diversity, *Lactobacillus*-dominated communities toward more taxonomically diverse, ARG-harbouring taxa. Increased redundancy was associated with higher microbial alpha diversity and enrichment of glycan-related metabolic pathways, particularly UDP-N-acetyl-D-glucosamine biosynthesis, a precursor pathway for glycan assembly. These microbial functional features correlated with host glycomic signatures in serum and urine. Mediation and path modelling analyses supported associations among resistome FR, predicted UDP-sugar pathway potential, host glycomic variation, and endocrine-related traits. Our findings identify resistome FR as a quantifiable ecological property of the vaginal microbiome that is associated with microbial metabolic capacity and host glycomic variation in endometriosis. Rather than acting as a direct driver, FR may serve as a marker of microbiome organisation that relates to host-associated phenotypes, providing a framework for understanding microbiome–glycome interactions in reproductive tract disease.

## Introduction

Understanding the structure and dynamics of microbial communities is crucial for unravelling their influence on host health^1–3^. Studies across systems—from gut^4–6^ to reproductive tract^7^—have linked microbiome composition to host phenotypes. However, taxonomic shifts do not always align with clinical outcomes. As a chronic gynaecological disease, endometriosis is a major cause of infertility and is associated with increased risks of cancer and autoimmune diseases^8,9^. Endometriosis patients exhibit a distinct vaginal microbiota characterised by loss of dominant *Lactobacillus* spp. (i.e., *L. iners*) and enrichment of anaerobes such as *Anaerococcus senegalensis* and *Prevotella jejuni*^8^. Yet these compositional differences alone do not fully explain host phenotype severity^8^. Taxonomic profiles show which microorganisms are present, but not what functions they encode or have (in)activated. Transcriptomics can address this gap, but it is challenging in low-biomass samples such as vaginal swabs. Thus, alternative strategies to examine the microbiome’s functional potential, particularly at the gene level, become critical to understand how microbial shifts relate to host phenotypic variations.

Antibiotic resistance genes (ARGs) are widespread and shift with microbial composition^10–12^. While ARGs are traditionally studied in the context of antimicrobial resistance, they may also reflect broader ecological and functional properties of microbial communities. Rather than directly mediating metabolic processes, the resistome may act as a proxy for underlying community structure, functional capacity, and ecological interactions. In microbial ecosystems, functional redundancy (FR) describes the extent to which multiple taxa encode overlapping functional traits and is often associated with community stability and adaptation under environmental or host-associated pressures^13–17^. This redundancy buffers ecosystem functions against species loss^18,19^. Our previous study on infant stool samples identified factors influencing FR of the resistome (i.e., the collective set of ARGs)^20^. However, how redundancy links microbial community structure to host phenotypic variations, particularly under low-biomass conditions (i.e., in the reproductive tract), remains unclear. Hence, we propose a functional framework to investigate the role of the resistome in host-microbiome interactions in endometriosis.

In addition to vaginal microbial alterations, endometriosis is associated with changes in host immune signalling, glycosylation, and endocrine function^8^. Our prior work revealed that host glycomes are related to endometriosis severity; specifically, serum trigalactosylated and triantennary *N*-glycans, as well as urinary IgG core fucosylated mono-antennary *N*-glycans, were correlated with the vaginal presence of *A. senegalensis* in affected patients^8^. This observation suggests that vaginal microbial perturbations may extend beyond compositional shifts to reflect glycan-associated signalling processes in the host. However, it remains unknown whether functional variation within the vaginal resistome relates to microbial metabolic potential in ways that interact with host glycosylation pathways and downstream inflammatory or endocrine phenotypes.

Here, we test the hypothesis that resistome FR of the vaginal microbiome links microbial composition to host metabolic and clinical variation in endometriosis. We highlight resistome redundancy as an ecological marker of microbiome organisation that is associated with microbial metabolic capacity and host glycomic variation, rather than a direct mechanistic driver of host phenotypes. Using deep shotgun metagenomics, quantitative glycomic profiling, and statistical modelling, we characterise the vaginal resistome across endometriosis severity, assess its relationship with microbial metabolic pathway capabilities, and evaluate how these microbial functions connect to host glycomic features and reproductive phenotypes.

## Methods

### Study design

The Research Ethics Committee of the National Maternity Hospital, Dublin (Ref. No.:EC19.2018) approved this protocol. Written informed consent was obtained from all participants. This research was performed in accordance with the Declaration of Helsinki. All patients underwent laparoscopy for the investigation of infertility, pelvic pain or pelvic pathology suspected on ultrasound scan. Patients were separated into controls (19), minimal/mild (11), and moderate/severe (10) endometriosis. The severity of endometriosis was classified according to the revised American Society for Reproductive Medicine classification^21^. None of the women had been using any hormonal medications and/or anti-inflammatory therapy for at least 3 months prior to surgery. All women had negative screening results for sexually transmitted diseases, active systemic infection, a history of autoimmune diseases, active vaginosis, acquired or primary immunodeficiency diseases (including HIV), pregnancy, or a malignant condition.

### Sample collection

High vaginal swabs were collected by laparoscopy. Some patients who did not have a vaginal swab taken at the time of laparoscopy subsequently had this performed as an outpatient in the mid-luteal phase of the cycle. Samples were preserved in DNA Genotek OMNIgene preservative.

### DNA isolation and sequencing

Details regarding nucleic acid extraction and metagenomic sequencing procedures have previously been reported^8^. DNA was extracted from high vaginal swab samples using an optimised bead-beating protocol with variable-size microbeads, combined with freeze–thaw cycles at −80 °C to enhance lysis efficiency and overcome connective tissue resistance. Sample preparation was performed under a laminar flow hood using the QIAamp UCP Pathogen Mini Kit (Qiagen), following the manufacturer’s instructions. Extracted DNA was quantified using a Qubit 4 Fluorometer (Thermo Fisher Scientific), and microbial load was assessed by 16S rRNA-targeting qPCR. Samples were stored at −80 °C until sequencing and shipped on dry ice to CosmosID Inc. (Germantown, MD, USA) for whole-genome shotgun sequencing. Sequencing libraries were prepared using the Nextera XT DNA Library Preparation Kit (Illumina) with IDT Unique Dual Indexes. Libraries were sequenced using the NovaSeq 6000 System (Illumina) with 150 bp paired-end reads. Positive and negative controls, as well as technical replicates, were included for both extraction and sequencing; all passed quality control. The number of raw sequence reads per sample ranged from 36.9 M to 96.2 M (mean = 63.3 M), whereas control glove samples yielded 0.48 M reads, mostly matching human and skin microbiota.

### Identification of ARGs from metagenomic reads

The raw metagenomic reads were subjected to quality control using Cutadapt (v 2.6)^22^ and FastQC (v 0.11.8)^23^. Trimmed reads were aligned to the human genome using Bowtie2 (v 2.5.1)^24^, aligned reads were removed with Samtools (v 1.17)^25^, and the remainder were converted to quality-controlled microbial FASTQ reads. The ARGs online analysis pipeline (ARGs-OAP v 3.0) was used for resistome analysis^26,27^. The Structured Antibiotic Resistance Gene (SARG) database identifies ARG types (corresponding to an antibiotic class) and subtypes within each class. Diversities are hence defined as the number of subtypes obtained for each ARG type^28^. The parameters used for ARG identification were alignment length cut-off of 75 nucleotides, alignment *e*-value at 1 × 10^-10^, and alignment identity of at least 80%^28^. ARG abundances were then normalised by the 16S rRNA gene as previously described^26–29^ using the following equation:

$${Abundance}=\mathop{\sum }\limits_{1}^{n}\frac{{N}_{{ARG}-{Like}{Sequence}}\,\times \frac{{L}_{{reads}}}{{L}_{{ARG}{Reference}{sequence}}}}{{N}_{16S{sequence}}\,\times \frac{{L}_{{reads}}}{{L}_{16S{sequence}}}}$$

The average abundance (i.e., the mean of the abundances across all samples in the dataset) >0.001 was considered as the detection threshold of antibiotics and ARGs^30^.

### Identification of ARG-encoding bacterial hosts and resistome-microbiome networks

Metagenomic reads identified as encoding ARGs were first extracted and converted into FASTA format. These reads were then compiled into a multiFASTA file, which served as the reference for aligning quality-controlled metagenomic reads from each sample using Bowtie2^24^. Subsequently, Samtools (v 1.17)^25^ was used to convert the aligned SAM results to BAM format. Only mapped reads were extracted and sorted. Finally, fastq files containing reads that were potential genomic carriers of ARGs were generated. These reads were then classified with Kraken2 using the NCBI bacteria database (accessed November 2025) with a confidence score setting to 0.1^31^, and the relative abundance of taxa was calculated at phylum, class, genus, and species levels using Bracken^32^. Only 34 samples (controls = 15, minimal/mild = 9, moderate/severe = 9) had sufficient reads to allow taxa assignment to species-level by Kraken2/Bracken. Samples without reliable species-level assignments (e.g., reads classified only at higher taxonomic ranks) were excluded from species-level analyses. Networks based on Spearman correlations (*P*_FDR-adjusted_ < 0.05, *ρ* > 0.6) were used to identify co-occurrence networks between the relative abundance of ARG-harbouring bacteria and ARG abundance.

### Calculation of resistome functional redundancy in the vaginal microbiome

Functional redundancy (FR) of a defined microbial community is considered to be part of the taxonomic diversity (TD) that cannot be explained by the functional diversity (FD), indicating that functional redundancy is calculated as the difference between TD and FD^15,33^. The Gini-Simpson index (GSI) was taken as an indicator of TD, and FD was calculated as Rao’s quadratic entropy (*Q*)^34^. The functional redundancy of ARG-harbouring bacteria was calculated using the following equation: functional redundancy = GSI – *Q,* and ranges from 0 to 1. When functional redundancy = 0, it indicates that species are completely different in their functions, and when all the species are functionally identical, the functional redundancy of a microbial community tends to be 1^34^.

To compute FR, microbial community composition was obtained as relative abundance tables at the species level for each sample. Taxa with zero abundance across all samples within each group were removed prior to analysis. To define functional traits, we constructed ARG–microbe association matrices for each group. Specifically, Spearman correlation analysis was performed between ARG abundance profiles and microbial taxa abundances with Benjamini–Hochberg (BH) correction applied for multiple testing. Significant associations were defined as those with adjusted *P* < 0.05. Based on these significant correlations, a binary ARG–microbe association matrix was generated, where rows represent ARGs and columns represent microbial taxa, with values of 1 indicating a significant association and 0 otherwise. This matrix was then transposed to define the trait matrix, in which each microbial taxon is characterised by its associated ARG profile. The FR of ARG content within ARG-harbouring bacteria was then calculated using the ‘rao.diversity’ function in the ‘SYNCSA’ package in R^35^. The community matrix (comm) consisted of microbial relative abundances for each sample, and the trait matrix (traits) represented ARG profiles associated with each taxon. FR was treated as a continuous variable in all statistical models.

Given the ecological characteristics of the vaginal microbiome, which is typically dominated by a single species (most commonly a member of the genus *Lactobacillus*) and exhibits low taxonomic diversity, we also classified samples into two categories based on FR: FR = 0 and FR ≠ 0. This classification reflects two distinct ecological states. Samples with FR = 0 represent functionally non-redundant communities, in which a dominant taxon carries most of the functional traits, resulting in minimal overlap across taxa. In contrast, samples with FR ≠ 0 represent functionally redundant communities, characterised by the co-occurrence of multiple taxa sharing overlapping functional traits. This classification is only for ecological interpretation, rather than representing an arbitrary dichotomisation of a continuous variable or statistical inference.

### Relationships between functionally redundant resistome and vaginal microbial community structures

Alpha diversity indices, including Chao1, Shannon, and Simpson, were calculated previously^8^. Differences in alpha diversities between redundant and non-redundant groups were assessed using Wilcoxon rank-sum tests. Beta diversity was evaluated using Bray–Curtis dissimilarities and ordinated through principal coordinate analysis (PCoA). To examine associations between resistome redundancy and host phenotypes, comparisons of alpha diversities across clinical groups within redundant groups were performed using Kruskal–Wallis tests. Linear regression models were applied to assess the relationship between FR values and alpha diversity indices for redundant samples.

### Quantification of microbial metabolic pathways and association with resistome redundancy

High-quality metagenomic reads were translated and aligned against the UniRef90 protein database (accessed November 2025) using a translated search. Gene family abundances were computed^36^, which weights read mapping by alignment quality, coverage and gene length. UniRef90 gene families were then functionally annotated to MetaCyc reactions, enabling the reconstruction and quantification of MetaCyc metabolic pathways based on reaction-level enzyme abundance.

To identify microbial functions associated with resistome FR, we first compared MetaCyc pathway abundances between non-redundant and redundant communities. For each pathway, Spearman correlations were calculated between FR values and log-transformed pathway abundances (FDR-adjusted *P*-values). Pathways with *P* < 0.05 were considered FR-associated and were used for visualisation.

### Associations between microbial metabolic pathways and host glycomes

Host glycome was previously measured in serum IgG, whole serum, urine IgG and whole urine, where glycans were released using PNGase F and analysed via UPLC/mass spectrometry^8^. We then examined associations between the two UDP-GlcNAc biosynthesis pathways and quantitative glycan traits. Spearman correlation coefficients (*ρ*) were calculated across all samples with complete data (FDR-adjusted *P*-values). Glycans showing *P* < 0.05 were considered as candidate pathway-linked glycans.

### Assessment of the contribution of functional redundancy independent of microbial diversity

To assess whether FR provides information beyond overall microbial diversity, we performed multivariable regression analyses including both FR and Shannon diversity as predictors of UDP-sugar pathway abundance. Shannon diversity was selected as a representative alpha-diversity metric as it captures both richness and evenness and is widely used in microbial ecology. For each UDP-sugar biosynthesis pathway, we fitted linear models with log1p-transformed pathway abundance as the dependent variable. Models including Shannon diversity alone were compared to models including both Shannon diversity and FR using analysis of variance (ANOVA) to evaluate the additional explanatory value of FR. All numeric predictors were standardised (*z*-scores) prior to modelling to allow direct comparison of effect sizes.

### Multivariable modelling of microbial UDP-sugar biosynthesis pathways

To further quantify the independent association of FR to UDP-sugar metabolism, we fitted linear regression models for UDP-N-acetyl-D-glucosamine biosynthesis pathways I and II, separately. For each pathway, abundances were log1p-transformed to reduce skewness and used as the dependent variable. Candidate predictors included FR and a set of 12 clinical and biochemical covariates (Supp 1). All numeric predictors were standardised (*z*-scores) prior to modelling. For each pathway, we specified a null model and a full model including FR and all available covariates. We then performed bidirectional stepwise selection based on Akaike’s information criterion (AIC, MASS package in R). The final model for each pathway was used to obtain standardised regression coefficients (*β*), and 95% confidence intervals via the parameters package, and partial *R*² values via effect size.

### Mediation analysis of FR, UDP-GlcNAc pathways, and host phenotypes

To explore potential relationships between FR, microbial UDP-sugar pathways, and host phenotypes, we performed mediation analysis. For each of the clinical and endocrine variables, FR was specified as the treatment (exposure), the UDP-GlcNAc biosynthesis pathway (I or II, log1p-transformed) as the mediator, and the phenotype as the outcome. Analyses were restricted to continuous clinical and endocrine variables appropriate for linear modelling. Samples with missing values in any of the variables included in a given model were excluded on a per-phenotype complete-case basis, resulting in variable sample sizes across analyses. For each phenotype and each pathway, we fitted a mediator model (Pathway ~ FR) and an outcome model (Outcome ~ FR + Pathway) using linear regression. Non-parametric bootstrapping (5000 simulations) was used to estimate the average indirect effect (ACME), average direct effect (ADE), and total effect (‘mediation’ package in R). Resulting estimates (indirect, direct, and total effects) were interpreted as statistical associations rather than causal effects. To account for multiple testing across phenotypes, *P*-values were adjusted using the Benjamini–Hochberg method.

### Modelling of relationships among resistome FR, microbial UDP-sugar metabolism, glycome variation, and host phenotypes

To integrate FR, microbial UDP-sugar metabolism, glycome variation and host outcomes into a unified structural framework, we applied partial least squares path modelling (PLS-PM, plspm package in R). Four constructs were defined: FR, represented by a single observed variable (resistome FR); Pathway, defined as a latent construct measured by two indicators: UDP-sugar biosynthesis pathways I and II; Glycome, defined as a latent construct composed of selected glycan features with moderate-to-high outer loadings; and Phenotype, defined as a latent construct based on clinical and endocrine variables. The structural (inner) model specified directional relationships among constructs (FR → Pathway → Glycome → Phenotype), while allowing additional direct paths. Model parameters were estimated using standardised data. Statistical significance of path coefficients was assessed using bootstrapping with 5000 resamples. Outer loadings (*λ*) quantify the strength of association between observed variables and their corresponding latent constructs. Path coefficients (*β*) represent standardised relationships between constructs. *P*-values were derived from bootstrap distributions. *R*² values indicate the proportion of variance explained in each endogenous construct.

## Results

### Vaginal resistome composition varies with endometriosis severity

**Fig. 1:**
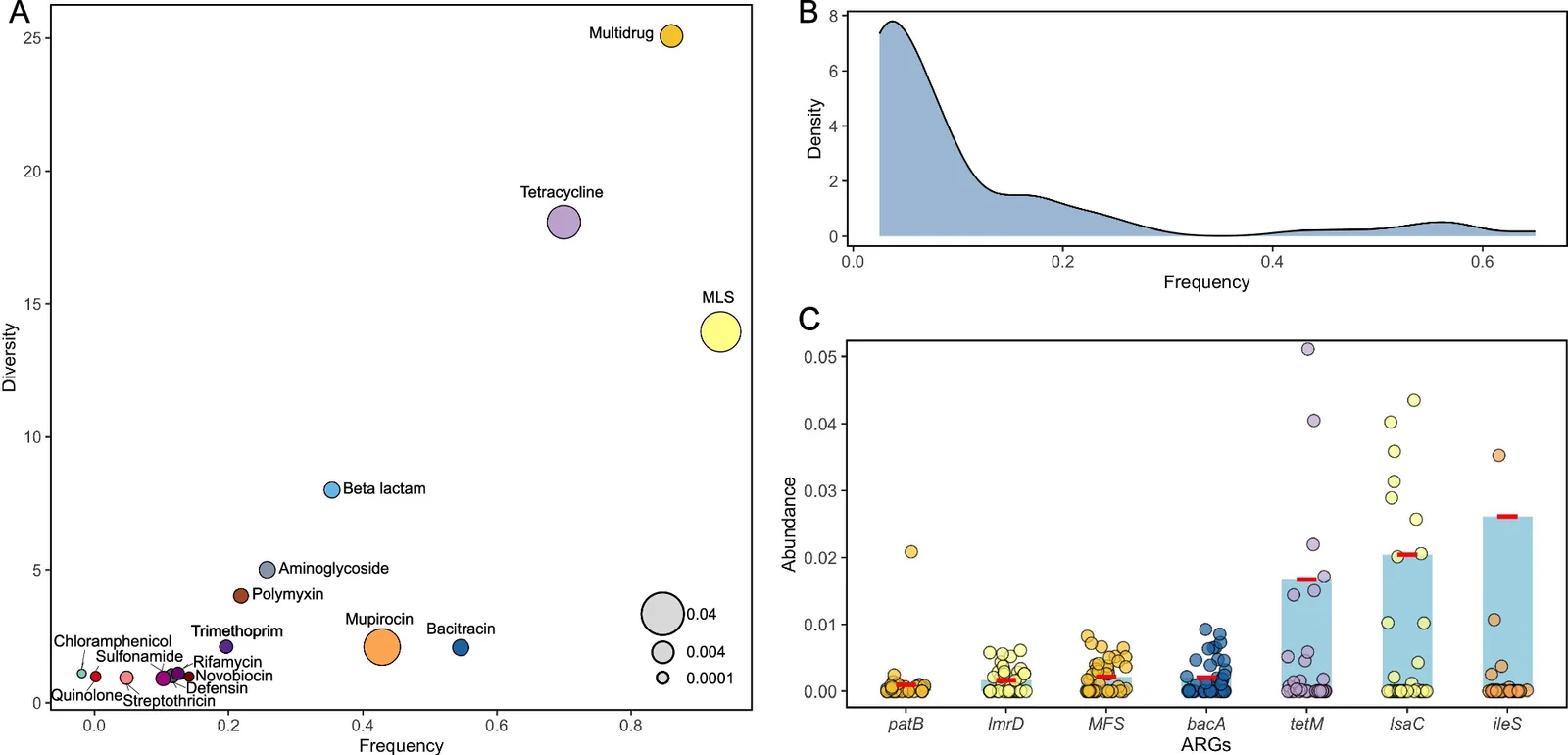
ARG profiles present in vaginal swab samples across healthy control (*n* = 19), minimal/mild (*n* = 11) and moderate/severe cases (*n* = 10). **A** Frequency and diversity of antibiotic classes. The prevalence (*x*-axis) refers to the number of vaginal swab samples containing an ARG predicted to encode resistance to each class of antibiotic, and the diversity refers to the number of ARG subtypes belonging to each antibiotic class. The size of each dot is proportional to the abundance of each antibiotic class. *n* = 40. **B** Distribution of ARGs carried by vaginal bacterial species. The frequency refers to the number of ARGs encoded by a bacterial species. **C** Relative abundance of the seven most abundant ARGs across all samples regardless of host phenotypical variations.

After host DNA removal, samples retained an average of 3.85 ± 0.29 million microbial reads (range: ~1.07–9.61 million), supporting sufficient sequencing depth for resistome profiling. A cohort of 40 participants was recruited and categorised into three groups: controls (*n* = 19), minimal/mild (*n* = 11), and moderate/severe (*n* = 10) endometriosis. High vaginal swabs were collected and subjected to shotgun metagenomic sequencing. We assessed the vaginal ARG profiles regardless of host phenotypes and assigned the identified resistance functions to a total of 17 antibiotic classes, involving 88 distinct ARGs, with an average class-level prevalence of 11% (Supp 2). Among these, the macrolide–lincosamide–streptogramin (MLS) class was the most abundant (average relative abundance: 3%) and most prevalent, detected in 39 out of 40 participants (97.5%, Fig. 1A, Supp 3). Among the detected ARGs, seven genes—*lmrD* and *IsaC* from MLS, *patB*, and *Other major facilitator superfamily transporter* (MFS) from multidrug, *bacA* from bacitracin, *tetM* from tetracycline, and *ileS* from mupirocin—showed a prevalence greater than 40% across all participants (Fig. 1B, C). *PatB* was the most prevalent, detectable in 26 out of 40 participants (65%).

When we looked at endometriosis severity, we identified 63, 41, and 35 diverse ARGs in control, minimal/mild, and moderate/severe groups, respectively. The most prevalent ARGs, *ileS, lmrD*, MFS*, patB*, and *tetM* had decreased prevalence in the moderate/severe group, whereas *bacA* and *IsaC* were increased in the moderate/severe group (Fig. S1). *IleS* was significantly more abundant in both the minimal/mild and moderate/severe group compared to controls (*P* < 0.05; Fig. S1).

Specifically, a total of 25 ARGs were shared among controls, minimal/mild, and moderate/severe groups, whereas 23 ARGs were detected in the control group (Fig. S2A). Seven ARGs were observed only in the minimal/mild group (Fig. S2A), whereas another distinct set of seven ARGs was exclusively present in the moderate/severe group (Fig. S2A). Most ARGs in the control group were associated with multidrug resistance, whereas ARGs detected in minimal/mild and moderate/severe endometriosis participants were mainly linked to tetracycline resistance (Fig. S2B–D).

### Severe endometriosis is characterised by a taxonomically expanded repertoire of ARG-harbouring species

**Fig. 2:**
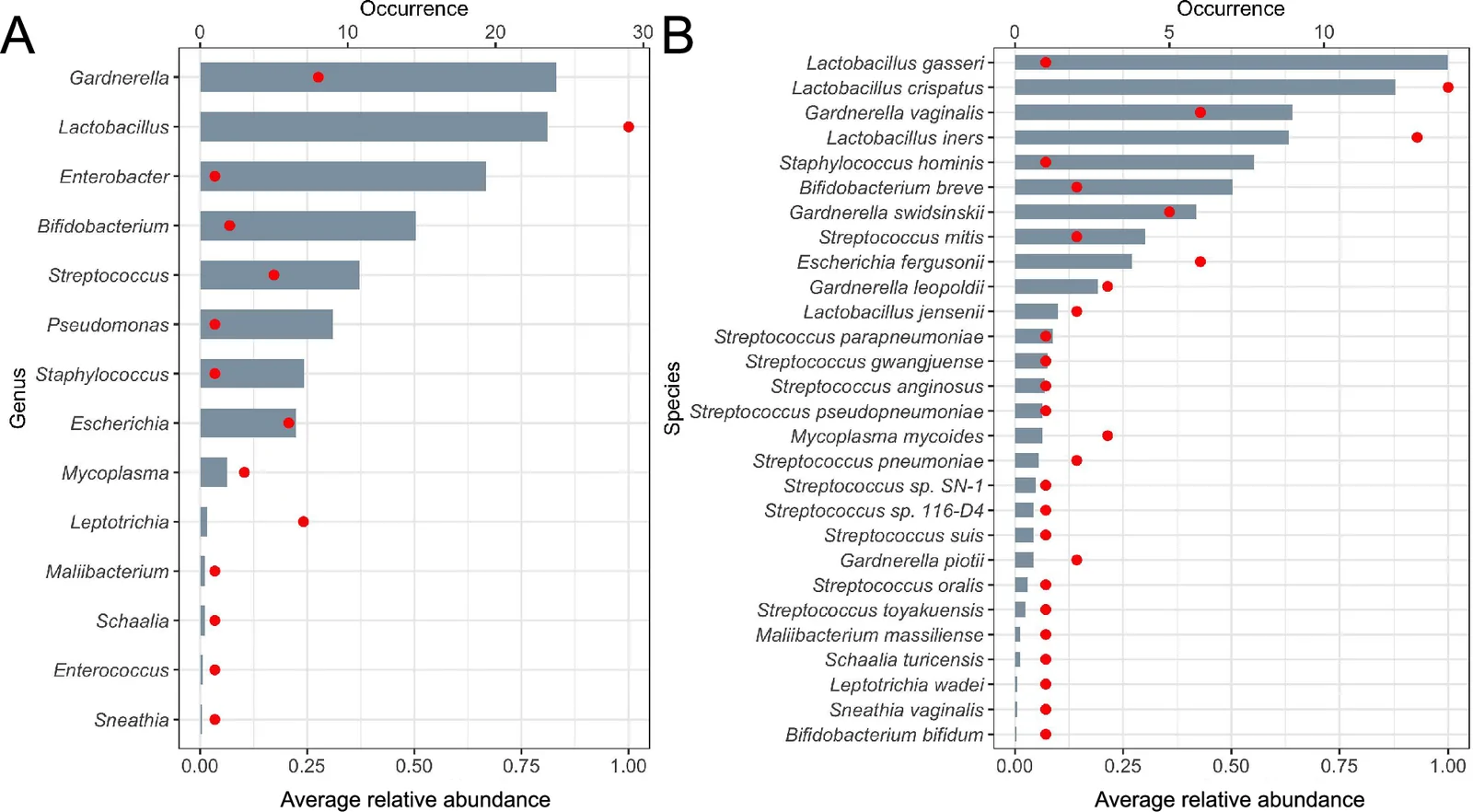
Taxonomic composition of ARG-harboring bacterial communities in the vaginal microbiome. Relative abundance of ARG-harbouring bacterial genera (**A**) and species (**B**) in vaginal microbial communities. Red dots indicate the occurrence of each genus (**A**) and species (**B**), with the corresponding axis shown at the top of the plot.

At the phylum level, six phyla were identified, including *Actinomycetota*, *Bacillota*, *Bacteroidota*, *Fusobacteriota*, *Mycoplasmatota*, and *Pseudomonadota*. Sixteen vaginal samples (controls: *n* = 8; minimal/mild: *n* = 5; moderate/severe: *n* = 3) were exclusively dominated by *Bacillota*. A total of 14 genera comprising 28 species were identified. At the genus level, *Gardnerella* and *Lactobacillus* exhibited the highest relative abundance regardless of host phenotypes (Fig. 2A). At the species level, although *Lactobacillus gasseri* exhibited the highest relative abundance, it was detected in only one participant, indicating that its dominance is not representative of the overall ARG-harbouring bacterial community (Fig. 2B and Supp 4).

Only two ARG-harbouring bacterial species were common to all groups, indicating that similar resistance functions may be carried out by different microbial taxa (Fig. S3 and Supp 4). Although the control group was found to exhibit the highest ARG diversity (63 ARGs), it had relatively fewer ARG-harbouring species (*n* = 8). In contrast, the moderate/severe group showed a moderate level of ARG diversity (46 ARGs), and the highest number of ARG-harbouring species (*n* = 24), suggesting a broader taxonomic distribution of ARGs in more severe cases. Fifteen ARG-harbouring bacterial species were exclusively detected in the moderate/severe group including *Lactobacillus gasseri*, *Streptococcus parapneumoniae*, *Gardnerella piotii*, *Streptococcus gwangjuense*, *Streptococcus pneumoniae*, *Streptococcus anginosus*, *Streptococcus pseudopneumoniae*, *Streptococcus* sp*. SN-1*, *Streptococcus* sp. *116-D4*, *Streptococcus oralis*, *Streptococcus toyakuensis*, *Maliibacterium massiliense*, *Schaalia turicensis*, *Leptotrichia wadei* and *Sneathia vaginalis*, nine of which belonged to the *Streptococcus* genus (Fig. S3).

### Functional redundancy increases with endometriosis severity and correlates with alpha diversity

We observed an increase in ARG-harbouring species without a corresponding increase in ARG diversity in moderate/severe endometriosis, consistent with functional redundancy, whereby multiple taxa carry similar resistance functions^3,13^. But it also suggests that the observed FR partly reflects expansion or diversification of ARG-harbouring taxa, particularly *Streptococcus* spp. By calculating the functional redundancy value, the value exhibited a trend for an increase in moderate/severe (0.24 ± 0.01), compared to control (0.12 ± 0.04) and minimal/mild (0.08 ± 0.05, *P* = 0.009). We noticed that 18 samples (controls: *n* = 7; minimal/mild: *n* = 6; moderate/severe: *n* = 5) exhibit functional redundancy values equal to 0, indicating a non-redundant ARG-harbouring community. Among the remaining samples with redundant communities (functional redundancy value > 0), we observed a significant increase in functional redundancy in the moderate/severe group (0.53 ± 0.13) compared to the control (0.22 ± 0.11) and minimal/mild (0.25 ± 0.15) groups (*P* = 0.03).

As part of the established cohort, vaginal microbiome analysis was performed in our previous study^8^. When comparing redundant and non-redundant communities—regardless of endometriosis severity—we observed a trend toward significantly higher alpha diversity (*P*_Chao1_ = 0.10, *P*_Shannon_ = 0.096, *P*_Simpson_ = 0.10) in redundant communities. Beta diversity analysis using PCoA revealed a separation between redundant and non-redundant communities (PERMANOVA pseudo-*F* = 2.72, *R*² = 0.081, *P* = 0.03; 999 permutations, Fig. S4). However, differences in within-group variability were also observed (*P* = 0.026, Fig. S4).

**Fig. 3:**
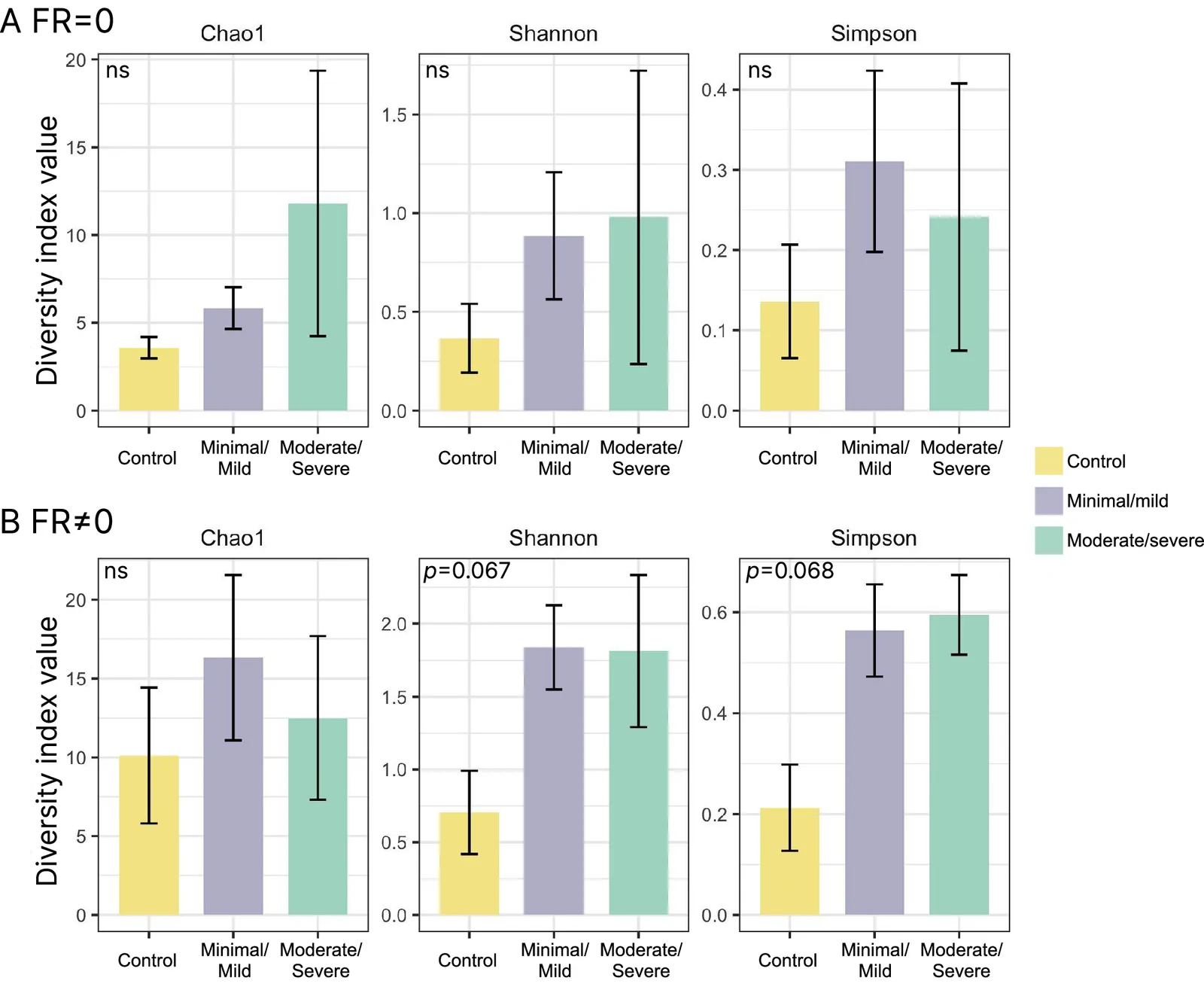
Alpha diversity of redundant and non-redundant vaginal microbial communities across disease groups. Comparison of alpha diversities within non-redundant (**A**) and redundant (**B**) microbial communities across healthy control, minimal/mild, and moderate/severe cases. **B** displays only samples with non-zero functional redundancy (Control *n* = 7; Minimal/Mild *n* = 6; Moderate/Severe *n* = 5). Box plots represent median and interquartile range (IQR); whiskers denote 1.5× IQR. Significance tested via Kruskal–Wallis test.

Incorporating host phenotypes into comparison, no significant differences in diversity indices were observed among clinical groups within the non-redundant communities (Fig. 3A). However, in redundant communities, we observed a tendency approaching significance in both Shannon and Simpson indices in minimal/mild and moderate/severe groups compared to controls (Fig. 3B). Linear regression analysis further confirmed that higher functional redundancy values were positively associated with increased alpha diversity (Shannon and Simpson, Fig. S5). Moreover, in the redundant community, both the resistome (*P* = 0.031, Fig. S6A) and the ARG-harbouring microbiota (*P* = 0.009, Fig. S6B) displayed significantly distinct compositional patterns across the three clinical groups.

### Distinct microbial taxa are associated with resistome redundancy in health versus severe endometriosis

**Fig. 4:**
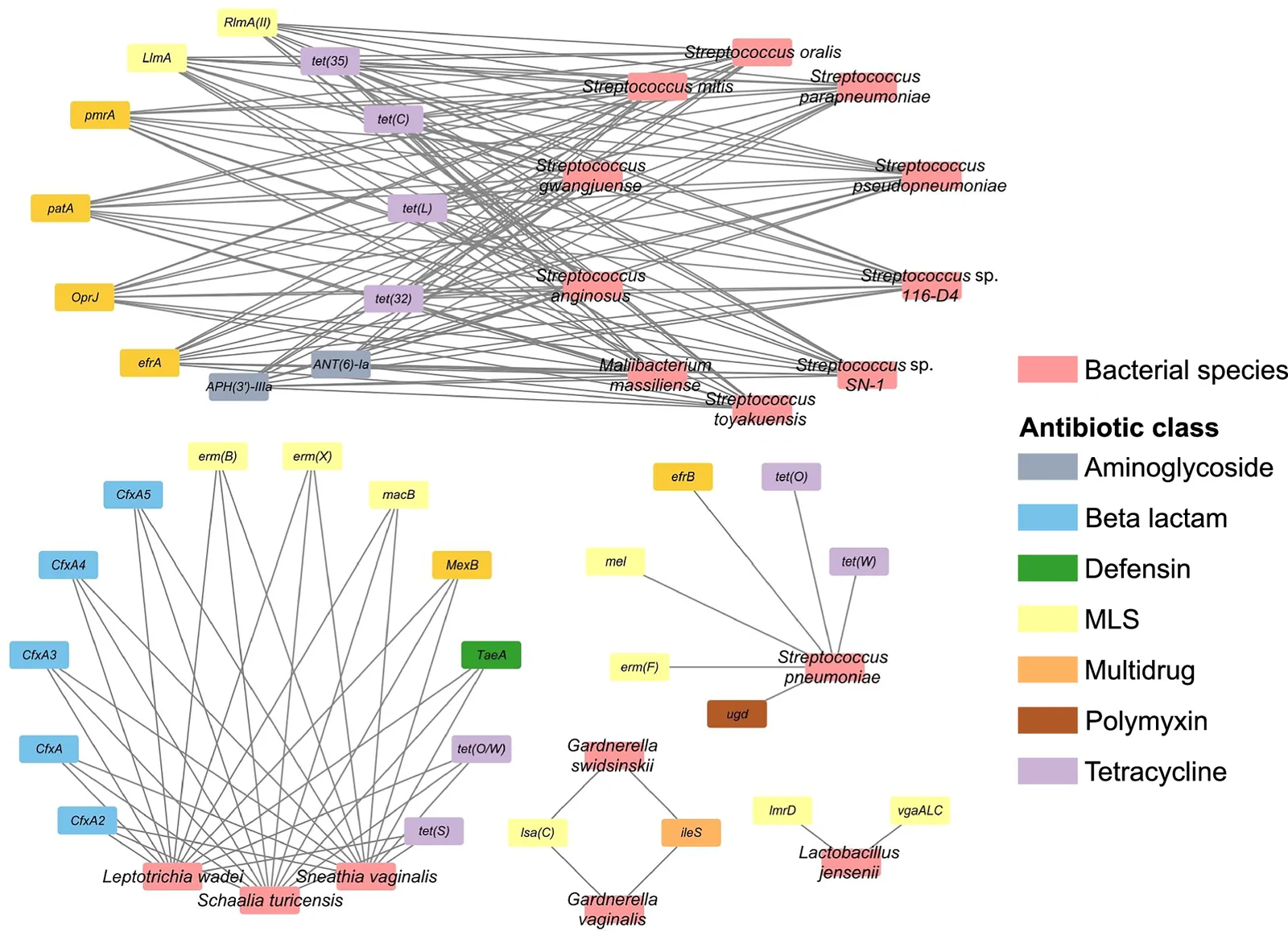
Co-occurrence network between bacterial species and ARGs in severe cases. The colour of each ARG refers to the class of antibiotic to which the gene is predicted to confer resistance. The network was constructed using Spearman correlations. Edges represent significant strong correlations (FDR-adjusted *P* < 0.05, | *ρ* | > 0.6).

Resistome–microbiome network analysis revealed that *Streptococcus mitis*, *S. suis*, and *Staphylococcus hominis* were the dominant species carrying 24 ARGs conferring resistance to eight antibiotic classes in controls (Fig. S7 and Supp 5). In contrast, the moderate/severe group was shown to exhibit a more diverse resistome–microbiome network, involving 17 bacterial species collectively harbouring 34 ARGs conferring resistance to eight antibiotic classes (Fig. 4 and Supp 5). Among the resistome–microbiome co-occurrence patterns in the moderate/severe group, 13 microbial taxa were observed only in this group.

### Microbial UDP-sugar biosynthesis pathways relate to resistome redundancy

**Fig. 5:**
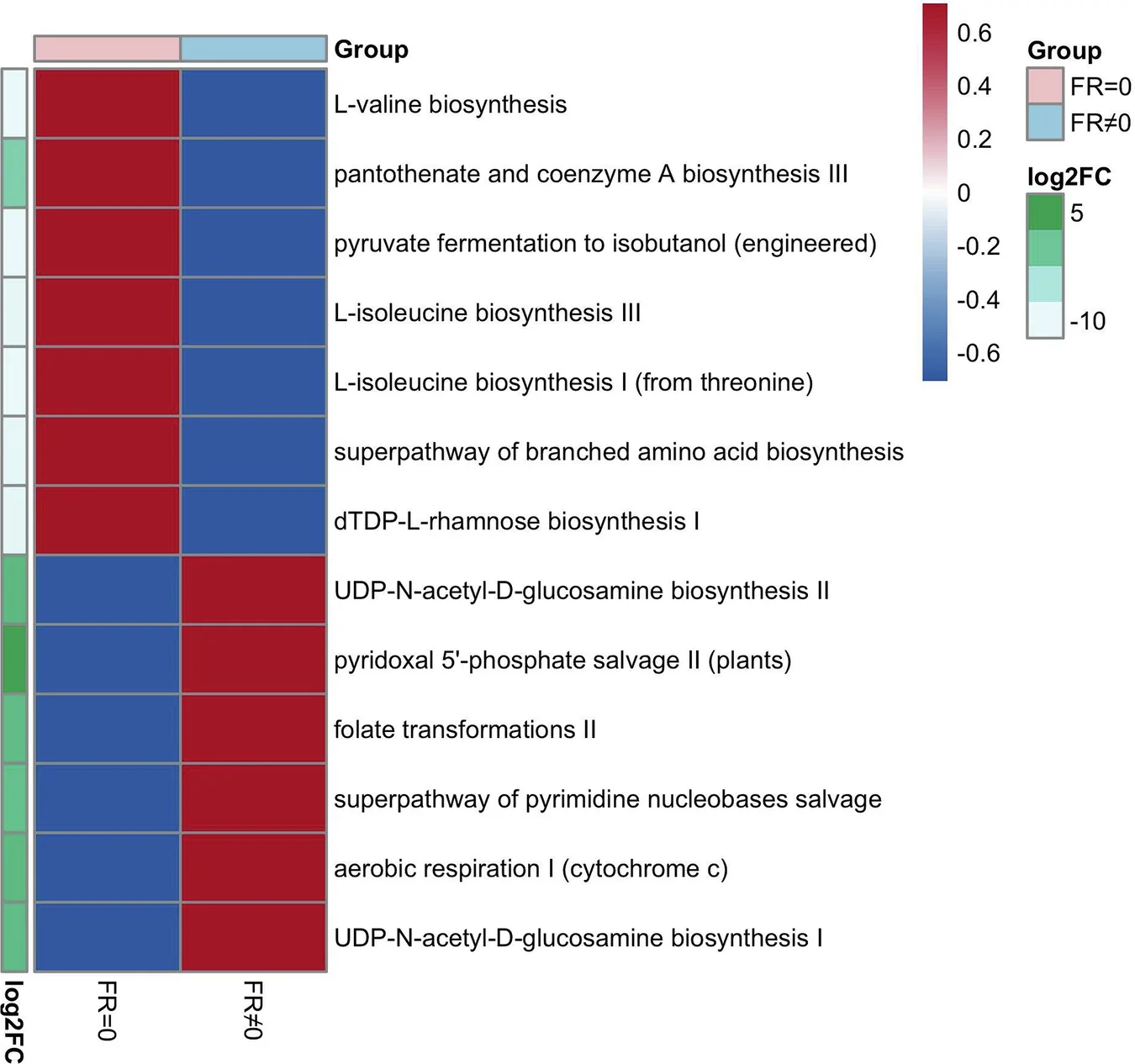
Differentially enriched metabolic pathways between functionally redundant and non-redundant groups. Log_2_ fold-change (log_2_ FC) of significantly differentially abundant MetaCyc metabolic pathways (FDR *P* < 0.05) between the two groups (FR = 0 and FR ≠ 0). Rows represent metabolic pathways, and columns represent the two groups. Red indicates higher relative abundance, whereas blue indicates lower relative abundance. The side colour bar indicates the magnitude of log2 fold-change for each pathway.

Comparing functionally redundant (FR ≠ 0) and non-redundant (FR = 0) communities revealed significant differences across multiple 13 metabolic pathways (Fig. 5 and Supp 6). Among these, UDP-N-acetyl-D-glucosamine biosynthesis pathway II showed the strongest positive association with FR (*ρ* = 0.57, FDR < 0.05). Notably, the related UDP-N-acetyl-D-glucosamine biosynthesis pathway I was also significantly associated with FR (*ρ* = 0.47, FDR < 0.05), indicating a consistent pattern across functionally linked pathways involved in nucleotide-sugar metabolism. UDP-sugar biosynthesis pathways directly encode nucleotide-sugar precursors (e.g., UDP-GlcNAc) that are central to glycan assembly in both microbial and host systems^37^. Since our previous findings had revealed that host glycome features are associated with endometriosis severity^8^, these pathways were prioritised for further analysis to explore their potential association with host glycomic variation.

To quantify the contribution of FR to microbial UDP-sugar biosynthesis pathways I and II in the context of host-related factors, we performed stepwise regression including FR together with host covariates. In stepwise regression models, FR was consistently retained in the final models for UDP-sugar biosynthesis pathways (I and II, Fig. S8). Standardised regression coefficients confirmed a robust positive association between FR and both pathways (UDP I: *β* = 0.50 [95% CI: 0.12–0.89]; UDP II: *β* = 0.54 [95% CI: 0.16–0.91]).

### Relationships between resistome redundancy and microbial UDP-sugar biosynthesis pathways are independent of microbial diversity

To determine whether overall microbial diversity contributes to resistome redundancy—microbial pathway relationships, we compared models including Shannon diversity alone with models including both Shannon diversity and FR. While Shannon diversity alone showed limited explanatory power for two UDP-sugar biosynthesis pathways (*R*²_UDP-I_ = 0.11, *R*²_UDP-II_ < 0.01), inclusion of FR significantly improved model fit (ANOVA, *F* = 9.22, *P* = 0.006). In multivariable models, FR remained positively associated with both UDP-N-acetyl-D-glucosamine biosynthesis pathways. For pathway I, FR showed a significant positive association (*P* = 0.027), whereas Shannon diversity did not retain explanatory power (*P* = 0.231). The model explained a moderate proportion of variance (*R*² = 0.305). Similarly, for pathway II, FR remained significantly associated with pathway abundance (*P* = 0.030), whereas Shannon diversity again showed no significant contribution (*P* = 0.488), explaining the proportion of variance (*R*² = 0.206).

### Microbial UDP-sugar biosynthesis pathways correlate with specific host glycome alterations

**Fig. 6:**
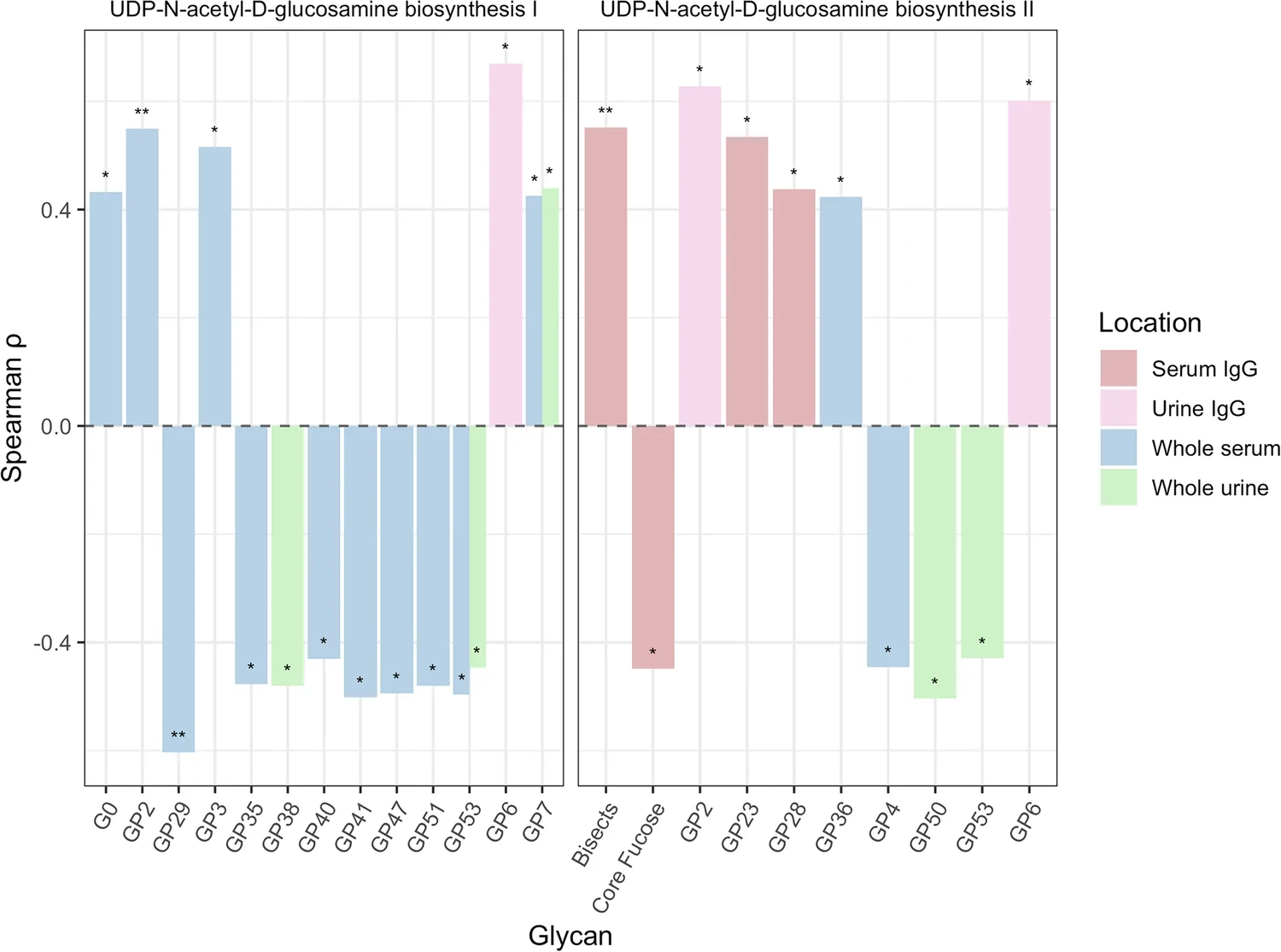
Correlations between glycan abundance and UDP-N-acetyl-D-glucosamine biosynthesis pathways. Spearman correlation coefficients (*ρ*) for significant associations (FDR *P* < 0.05) between individual glycans and the predicted abundances of UDP-N-acetyl-D-glucosamine biosynthesis I (left panel) and UDP-N-acetyl-D-glucosamine biosynthesis II (right panel). Each bar represents the correlation for a specific glycan, grouped by sample location: serum IgG (red), urine IgG (pink), whole serum (blue), and whole urine (green). Positive correlations (above the dashed zero line) indicate glycans positively associated with pathway abundance, whereas negative correlations (below the dashed line) indicate inverse associations. Asterisks indicate FDR-adjusted significance levels: FDR < 0.05*, FDR < 0.01**.

To investigate whether microbial functional pathways relate to host glycosylation, we examined the correlations between microbial UDP-sugar pathways and glycans and glycan traits spanning *N*-glycans from serum IgG, whole serum, urine IgG and whole urine glycoproteins (Fig. 6 and Supp 7). Whole serum glycoproteins separated into 56 glycan peaks (GPs)^38^, and urine glycoproteins, as well as urine and serum IgG GPs, were analysed as previously described^8^.

UDP-I showed clear associations with multiple glycan structures (Fig. 6), primarily within the whole glycoproteome-serum glycan (Fig. 6). Specifically UDP-I was positively associated with whole serum increases in G0 (agalactosylated glycans, *P* = 0.04), GP2 (core fucosylated monoantennary glycans, *P* = 0.006), GP3 (core fucosylated monoantennary glycans, *P* = 0.01), and GP7 (FA2, core fucosylated biantennary glycans, *P* = 0.04) but negatively associated with GP29 (biantennary digalactosylated disialylated glycans, *P* = 0.002), GP35 (triantennary trigalactosylated disialylated glycans, *P* = 0.02), GP40 (*P* = 0.04) and GP41(both triantennary structures, *P* = 0.01), GP47 (tetraantennary tetragalactosylated trisialylated glycans, *P* = 0.02), GP51 (tetraantennary glycans, *P* = 0.02) and GP53 (tetraantennary tetragalactosylated tetrasialylated glycans, *P* = 0.02). In the whole urine glycoprotein, UDP-I showed positive associations with GP7 (*P* = 0.04) but was negatively related to GP38 (*P* = 0.02) and GP53 (*P* = 0.04).

UDP-II showed a negative association with core fucosylation (*P* = 0.04) and a positive association with bisecting GlcNAc structures on serum IgG (*P* = 0.007). UDP-II was strongly associated with both serum IgG glycans, showing positive associations with GP23 (*P* = 0.01) and GP28 (*P* = 0.04, both core fucosylated bisected structures). For whole serum glycoproteins, UDP-II positively correlated with GP36 (bisected triantennary trigalactosylated disialylated glycans, *P* = 0.04) and negatively correlated with GP4 (biantennary glycans*, P* = 0.03). In urine, UDP-II was negatively associated with highly branched glycans (GP50 and GP53, both *P* < 0.05) and positively related to GP2 (core fucosylated monoantennary glycans, *P* = 0.03) and GP6 (core fucosylated bisected biantennary glycans, *P* = 0.04).

### Exploratory modelling reveals coordinated variation among resistome redundancy, UDP-sugar pathways, glycome features, and host phenotypes

To explore cross-domain associations among resistome redundancy, microbial UDP-sugar pathway potential, host glycomic features, and clinical phenotypes, we performed mediation analysis across 12 continuous clinical and endocrine variables. No statistically significant indirect associations were observed for either UDP-GlcNAc biosynthesis pathway I or pathway II after correction for multiple testing (Fig. S9), indicating that the current data do not support a robust indirect association between resistome FR and host phenotypes through UDP-sugar pathways. Although several phenotypes exhibited relatively large indirect effect estimates, including oestradiol, progesterone-related measures, and their ratios, these effects were not statistically significant (Fig. S9). These results therefore support an exploratory association structure, but do not establish a microbiome-to-glycome pathway independent of endometriosis severity or related clinical features.

**Fig. 7:**
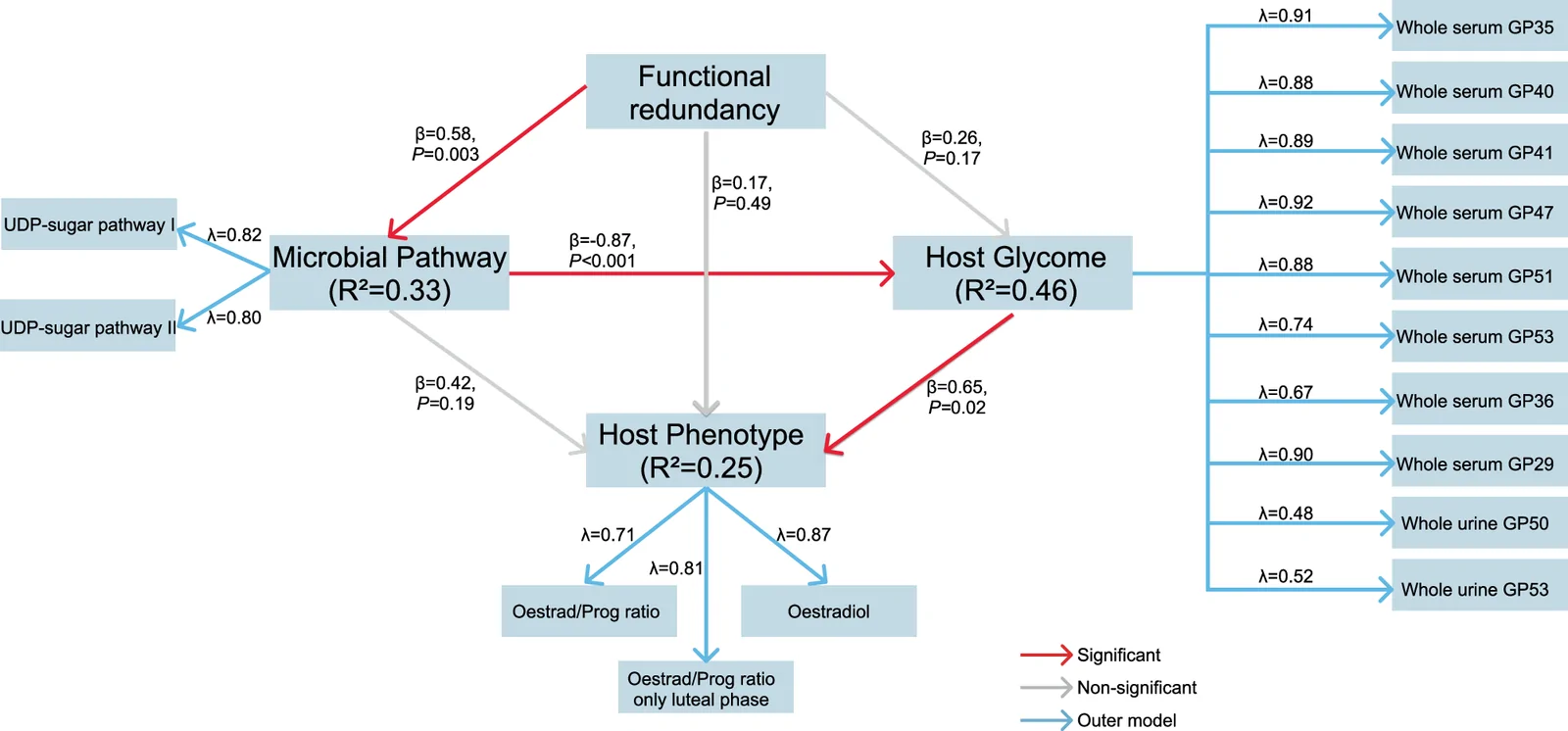
Partial least squares path model linking glycan profiles, microbial functional pathways, resistome redundancy, and host phenotypes. The diagram shows both the structural (inner) model and measurement (outer) model. Functional redundancy, microbial pathway, host glycome and host phenotypes represent latent constructs, and the rest represent observed variables. The pathway construct is defined by UDP-sugar biosynthesis pathways I and II. The glycome construct is composed of selected glycan features with moderate-to-high loadings, and the phenotype construct includes clinical variables. Numbers inside latent constructs indicate the coefficient of determination (*R*²), representing the proportion of variance explained by upstream variables. Arrows between constructs indicate standardised path coefficients (*β*), with associated bootstrap-derived *P-*values. Red lines represent statistically significant associations, while grey lines indicate non-significant relationships. Blue lines represent the outer model linking observed variables to their corresponding latent construct. Outer loadings (*λ*) represent the strength of association between indicators and their corresponding latent constructs.

To integrate microbial pathway potentials with glycomic features and host outcomes, we constructed a partial least squares path model (PLS-PM, Fig. 7). The model showed moderate explanatory performance (GOF = 0.50). FR showed a significant positive association with the pathway (*β* = 0.58, *P* = 0.003), explaining 33% of its variance (*R*² = 0.33). The pathway construct was strongly associated with glycome variation (*β* = −0.87, *P* < 0.001), collectively explaining 46% of glycome variance (*R*² = 0.46). Glycome variation was positively associated with host phenotypes (*β* = 0.65, *P* = 0.02), explaining 25% of phenotype variance (*R*² = 0.25). No statistically significant direct associations were observed between FR and glycome (*β* = 0.26, *P* = 0.17), FR and phenotype (*β* = 0.17, *P* = 0.49), or pathway and phenotype (*β* = 0.42, *P* = 0.19).

## Discussion

Our study explores associations among vaginal resistome structure, ARG-harbouring taxa, microbial metabolic potential, and host glycomic features in endometriosis. By integrating vaginal resistome and microbiome profiling with microbial pathway reconstruction, host glycomic measurements, and statistical modelling, we show that resistome FR reflects disease-associated microbial restructuring, particularly the expansion or diversification of ARG-harbouring taxa. Rather than indicating a resistome-specific mechanism, FR may serve as an ecological marker of how resistance-associated functions are distributed across vaginal microbial communities and how these patterns co-vary with microbial metabolic potential and host glycomic features.

We first show that the vaginal resistome is widespread but structured by disease severity: controls harbour the greatest diversity of ARGs but in a relatively small set of taxa, whereas moderate/severe endometriosis is characterised by a broader set of ARG-carrying species, particularly *Streptococcus* spp., without a proportional increase in ARG diversity. Several studies have reported enrichment of *Streptococcus* in endometriosis-associated microbial communities^39,40^. Therefore, the increase in ARG-based FR observed in moderate/severe endometriosis may partly reflect expansion or diversification of ARG-harbouring *Streptococcus* species, rather than a broad resistome-specific mechanism. In this context, FR should be interpreted as an ecological indicator of the distribution of resistance-associated traits across taxa within a dysbiotic community.

Our findings also suggest that progression towards more severe endometriosis is accompanied by a transition from low-diversity, functionally non-redundant resistomes to more taxonomically diverse, redundant ARG communities. This pattern is consistent with the ecological concept of functional redundancy^3,13,14^, in which microbial ecosystems under stress may undergo functional convergence, where diverse taxa encode similar functions to ensure resilience and adaptability^17,41^. Differences in sampling conditions, including collection during laparoscopy versus outpatient visits and potential variation in hormonal status, may represent confounding factors influencing resistome and functional profiles.

Functional redundancy is inherently function-specific: it describes whether a defined functional trait is encoded by one or several taxa within a community. We focused on ARGs because they provide a well-curated and reproducible functional domain, with class- and subtype-level annotations and broad distribution across microbial taxa^42^. Thus, ARG-based FR allows us to quantify how resistance-associated traits are distributed across vaginal microbial communities. This choice also helps maintain analytical separation between the redundancy metric and the downstream glycan-related analyses. Because our downstream analyses focus on microbial glycan-related pathways and host glycome features, we did not define FR using glycan-associated genes such as CAZymes or UDP-sugar pathway genes. Using these genes would make the FR metric overlap with the same biological processes examined later in the study. ARGs instead provide a distinct non-glycan cluster for assessing microbial community organisation from a redundancy perspective. Within this framework, redundant and non-redundant communities reflect distinct ecological states rather than data filtering. Non-redundant communities represent biologically meaningful low-diversity clusters. However, within redundant communities, ARG-harbouring taxa share overlapping resistance functions and contribute to a more redundant functional landscape. Therefore, ARG-based FR should be interpreted as redundancy within a defined resistome-associated functional cluster, rather than as total microbiome functional redundancy or evidence of a resistome-specific mechanism.

Moreover, functional redundancy in the vaginal resistome is sensitive to taxonomic scale. When calculated at the species level, FR captures fine-scale functional variation, whereas aggregation at higher taxonomic levels (e.g., genus) attenuated the signal by collapsing closely related taxa and reducing functional heterogeneity (At genus level: FR_control_ = 0.11, FR_minimal/mild_ = 0.02, FR_moderate/severe_ = 0.08). This effect is particularly relevant for antibiotic resistance genes, which are often shaped by horizontal gene transfer and can vary substantially even among closely related species^43^. These observations suggest that FR is a scale-dependent ecological property in the vaginal resistome that reflects the distribution of shared functions across taxa.

Methodologically, while assembly-based approaches can, in principle, enable the identification of ARG hosts, they are limited in resistome analyses due to the short, conserved, and mobile nature of ARGs, which increases the risk of fragmentation, misassembly, and collapse of homologous regions^44,45^, particularly in low-biomass environments with uneven taxonomic coverage. Due to the limitations of short-read metagenomics, including ambiguity in read mapping and horizontal gene transfer, ARGs cannot be reliably assigned to specific host taxa. Therefore, our interpretations are based on community-level associations rather than direct ARG–host linkage.

A key insight from this study is that vaginal resistome FR is associated with microbial glycosylation metabolism, particularly the UDP-sugar biosynthesis pathways. Our previous work in this cohort demonstrated that host glycome signatures differed across endometriosis severity, supporting glycosylation as a potential interface between the vaginal microbiome and host immune function^8^. Hence, we propose that ARGs can be used as a proxy for microbial community structure and functional capacity, rather than as direct mediators of glycosylation. In this context, functional redundancy captures how functions are distributed across taxa, which may co-vary with broader metabolic pathways encoded by the microbiome. Consistent with this, redundant communities showed enrichment of UDP-N-acetyl-D-glucosamine (UDP-GlcNAc) biosynthesis I and II pathways, which generate nucleotide-sugar precursors used in bacterial cell-wall and capsule assembly^37^, and are also central to host glycosylation processes. However, this association should not be interpreted as evidence that ARG carriage itself explains UDP-sugar metabolism. Because UDP-GlcNAc-related pathways contribute to streptococcal cell-wall, capsule, and cell surface-associated polysaccharide biosynthesis^46,47^, the FR–UDP-sugar association may partly reflect the metabolic repertoire of expanded ARG-harbouring taxa, particularly *Streptococcus* spp. Thus, ARG-based FR may capture disease-associated microbial restructuring that co-varies with glycan-related metabolic potential, rather than representing a resistome-specific mechanism.

We further observed correlations between UDP-sugar pathways and selected host glycome features. UDP-GlcNAc biosynthesis I and II showed significant correlations with a diverse set of serum IgG, whole serum and urine glycans. These correlations are biologically possible given that UDP-sugars, and UDP-GlcNAc in particular, are central donors for N-glycan branching and terminal modification reactions^37^. Because both microbial UDP-sugar pathway profiles and host glycome features may be associated with endometriosis severity or related endocrine and inflammatory states, these correlations should be interpreted as shared disease-associated patterns rather than evidence of a direct microbiome-to-glycome relationship. Systemic IgG glycosylation patterns are known to modulate Fc receptor binding and downstream inflammatory effector functions, with reduced galactosylation and sialylation and altered core fucosylation being repeatedly associated with chronic inflammatory diseases^48–50^.

By integrating resistome, metabolic, and glycomic data using the partial least squares path modelling (PLS-PM), we characterised relationships among these variables. Communities with higher redundancy were associated with increased pathway potentials and glycan profiles enriched in UDP-linked structures, several of which have established roles in immune regulation, inflammation, and epithelial homoeostasis^51,52^. The model supports coordinated variation among FR, microbial UDP-sugar metabolism, host glycomic variation, and host phenotypes, but does not establish a directional microbiome-to-glycome pathway or a causal indirect relationship.

While these findings suggest the relationship between vaginal microbial metabolic capacity and systemic glycomic features, the underlying mechanisms remain to be fully elucidated. One possible explanation is that glycosylation pathways are known to be sensitive to systemic inflammatory and endocrine states, which may be related to microbial-derived metabolites or immune mediators^53,54^. Alternatively, the observed associations may reflect coordinated variation driven by shared host factors, such as immune and/or hormonal status, which simultaneously shape both microbial community structure and host glycosylation patterns. Importantly, our data do not provide direct evidence for a causal relationship between vaginal microbiota and systemic glycosylation. Instead, these results highlight a consistent pattern of association that warrants further investigation using longitudinal designs and targeted measurements of immune mediators and metabolites.

These observations suggest that resistome redundancy may stratify microbial communities into distinct glycan-related functional potentials. Although the majority of taxa associated with high functional redundancy in this study were Gram-positive bacteria, which therefore lack the canonical UDP-GlcNAc II lipid A biosynthesis pathway, these organisms nevertheless exhibit substantial redundancy in UDP-GlcNAc-dependent structural glycan pathways. In particular, the *Streptococcus* species, together with *Lactobacillus gasseri*, *Schaalia turicensis*, and *Maliibacterium massiliense*, contribute to peptidoglycan, teichoic acid, and capsular polysaccharide biosynthesis, all of which generate potent TLR2- and NOD-mediated innate immune signals^55^. In parallel, three FR-associated Gram-negative taxa—*Gardnerella piotii*, *Leptotrichia wadei*, and *Sneathia vaginalis*—possess the complete UDP-GlcNAc II lipid A pathway, suggesting potential involvement in LPS-related processes^56^. Together, these observations suggest that FR-associated communities differ in predicted microbial structural glycan potential. These microbial features may co-vary with host glycome and inflammatory phenotypes within the broader disease-associated context, but they do not establish that resistome features directly shape host responses. These interpretations are based on inferred functional potential and require further experimental validation.

Several limitations should be considered in this study. First, the observed relationships between resistome redundancy, microbial metabolic potentials, and host phenotypes are based on cross-sectional, observational data, and therefore do not support causal inference. While our analyses identify consistent associations between microbial ecological structure, functional potential, and host glycomic and clinical traits, the underlying mechanisms of these relationships cannot be determined. In particular, resistome redundancy should not be interpreted as a direct driver of host metabolic or inflammatory processes, but rather as an emergent ecological property that reflects microbiome organisation. The associations observed here may arise from shared underlying factors, such as shifts in microbial community composition, host environment, or disease state, which jointly influence both microbial functional profiles and host physiology. The FR captures redundancy within one defined functional domain, the resistome, and should not be interpreted as total microbiome functional redundancy. Determining whether these associations are ARG-specific or reflect broader vaginal microbiome organisation will require comparison with other function-specific FR metrics. Future studies incorporating longitudinal sampling, strain-resolved metagenomics, and experimental validation will also be required to explore causal relationships and to determine whether resistome-associated microbial functions contribute directly to host glycomic variation and related phenotypes.

Second, as our analyses are based on metagenomic data, the inferred pathways represent functional potential rather than directly measured activity, and future studies incorporating transcriptomic, proteomic, or metabolomic data will be required to validate these findings. Also, direct measurements of immune mediators and microbial metabolites would be valuable to further evaluate these relationships.

Third, the relatively small sample size and multiple analytical steps may introduce a risk of overfitting. In addition, identified group-specific microbial taxa should be interpreted with caution, as they may be due to the limited sample size, detection thresholds and database limitations rather than true biological exclusivity. As such, regression-based model selection should be interpreted as exploratory, and the observed associations do not imply independent or causal effects. In addition, the current cross-sectional data cannot distinguish a direct microbiome-to-glycome relationship from shared associations with endometriosis severity or related clinical features. However, the key associations identified in this study were consistent across various analytical approaches, including group comparisons and multivariable modelling.

Across the spectrum of endometriosis severity, the vaginal resistome exhibits coordinated shifts in ARG composition, carrier taxa, and functional redundancy. Rather than indicating a direct causal relationship, the observed resistome features align with distinct microbial metabolic signatures—specifically the enrichment of UDP-sugar biosynthesis pathways—and with alterations in host glycosylation and inflammatory phenotypes. Collectively, these findings highlight resistome redundancy as an ecological marker of microbiome in the reproductive tract, providing a framework for understanding microbiome–host interactions in disease.

## Supplementary information

Supplementary Information

Supplementary Information

## Data Availability

Raw sequence data is available in the NCBI SRA under BioProject ID PRJNA1156592.
