# Supplementary material for: Vaginal resistome redundancy is associated with microbial metabolic potential and host glycomic features in endometriosis
Source: NPJ Biofilms Microbiomes. 2026 Jul 23;12:232. doi: 10.1038/s41522-026-01100-w (PMC13759476; doi:10.1038/s41522-026-01100-w)
Supplement: Supplementary file 1 [file 41522_2026_1100_MOESM1_ESM.pdf]

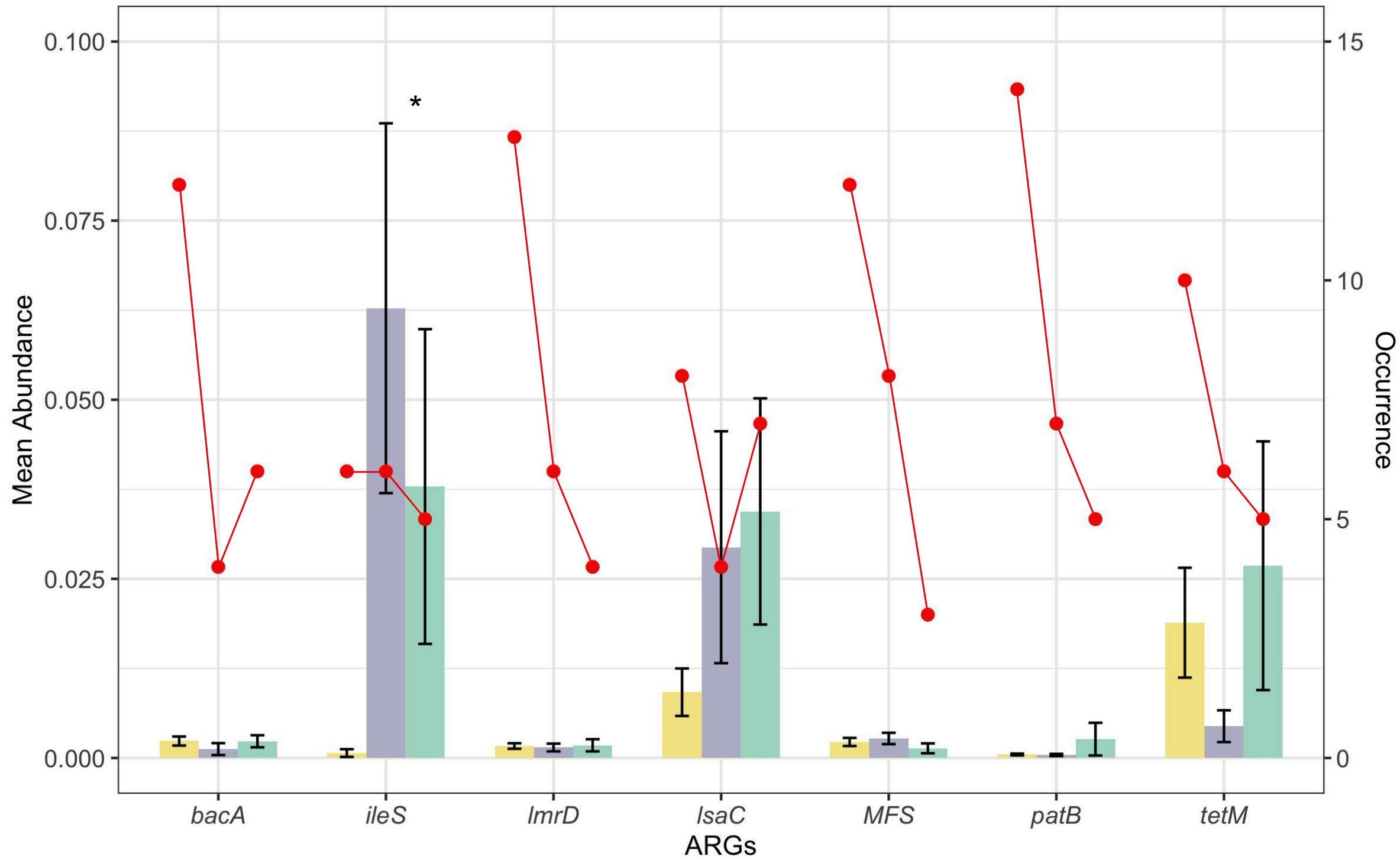

Fig S1. Mean abundance and occurrence of antibiotic resistance genes (ARGs) across sample groups.

Bar plots show the mean relative abundance of selected antibiotic resistance genes (ARGs) across sample groups, with error bars indicating standard error. Overlaid red lines and points represent the occurrence frequency of each ARG (right y-axis). Asterisks denote statistically significant differences in mean abundance between groups (*P* < 0.05).

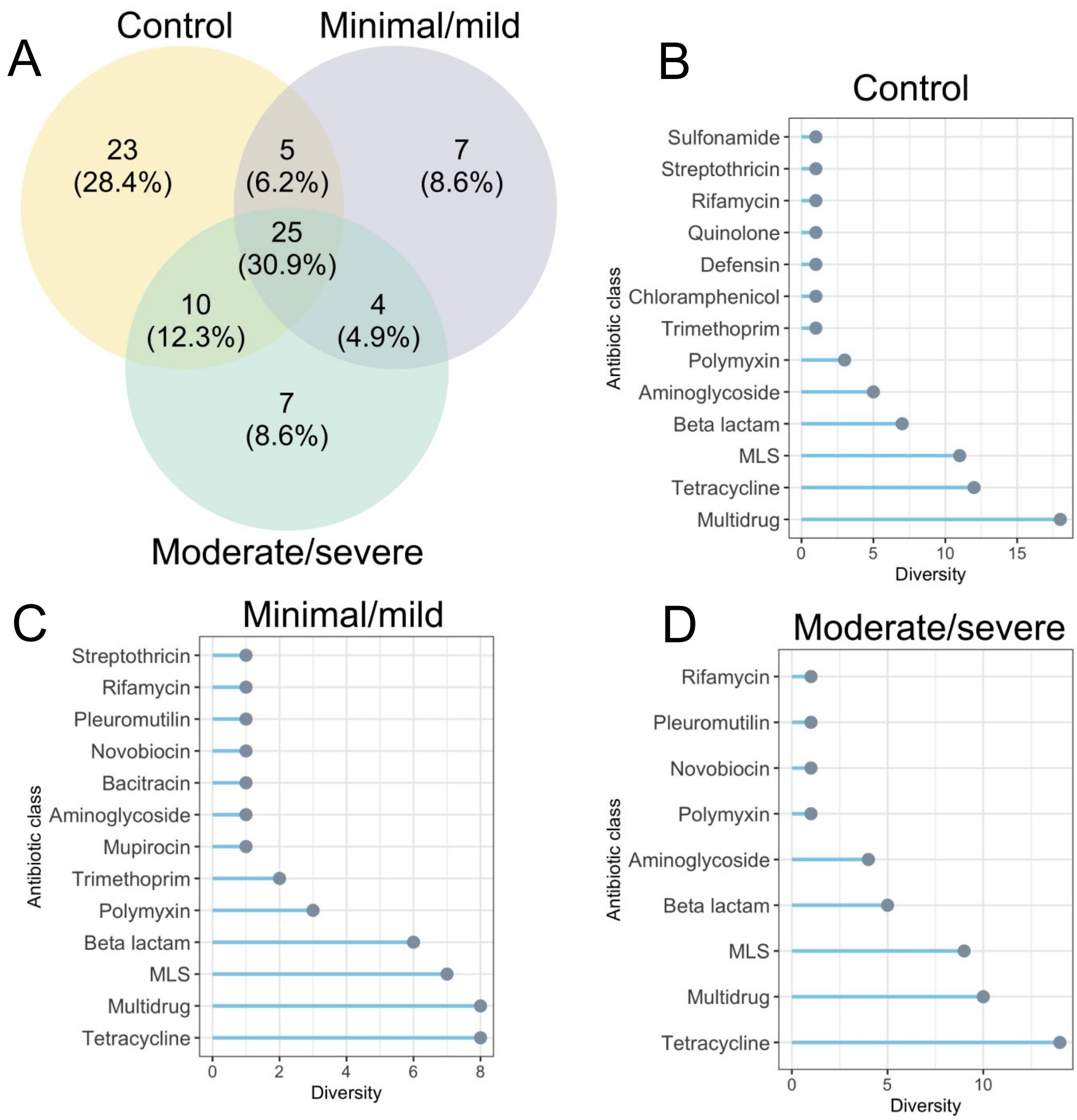

Fig S2. Comparative profiles of ARGs in vaginal microbial communities from healthy controls (n = 19), minimal/mild (n = 11), and moderate/severe cases (n = 10).

A. Venn diagram showing the shared and unique ARGs among healthy controls, minimal/mild, and moderate/severe cases.

B-D. Lollipop plots showing the antibiotic classes to which the ARGs belong in the control (B), minimal/mild (C), and moderate/severe (D) groups.

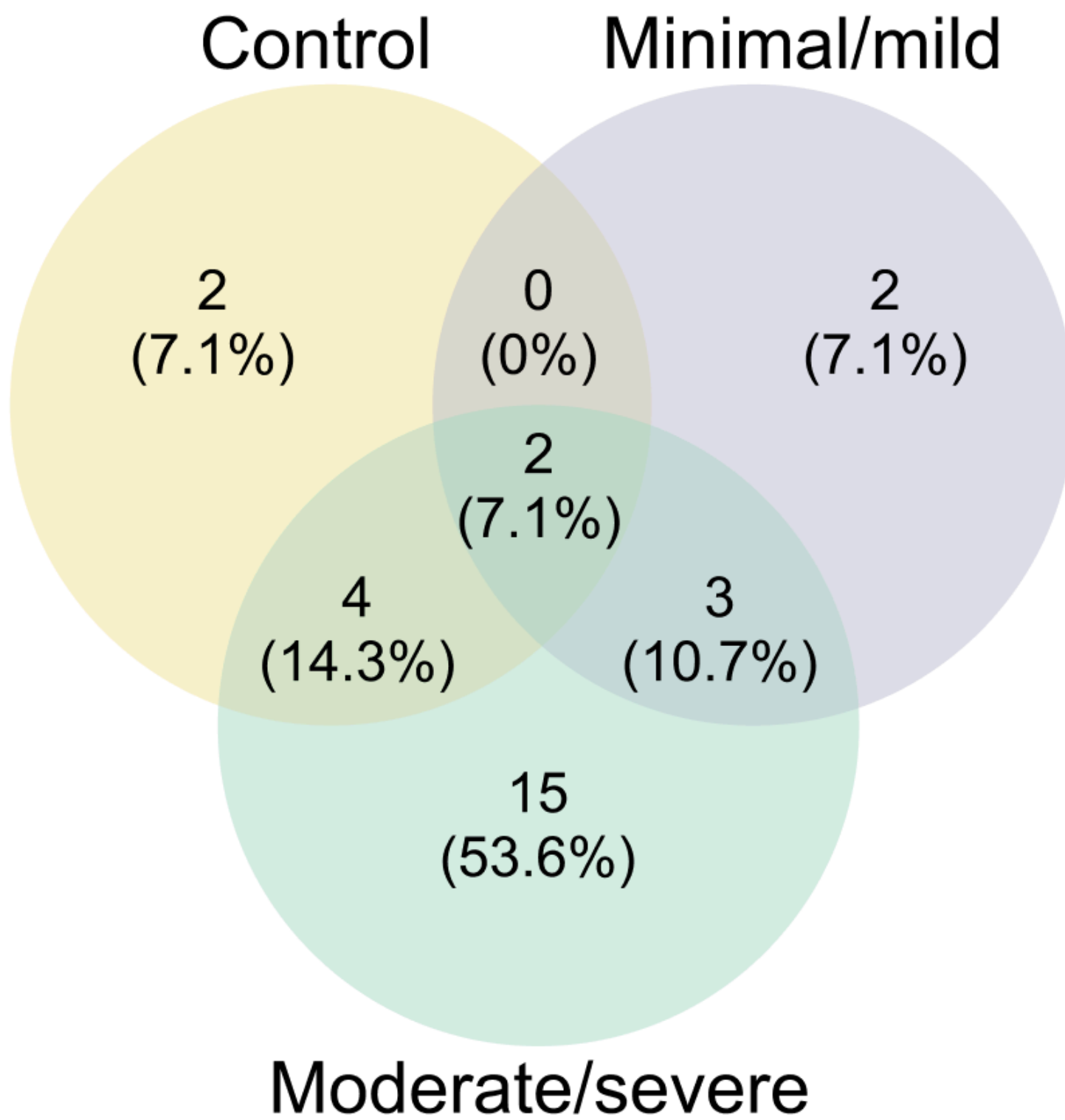

Fig S3. Venn diagram illustrating the shared and unique ARG-harboring bacterial species among healthy controls (n = 19), minimal/mild (n = 11), and moderate/severe cases (n = 10).

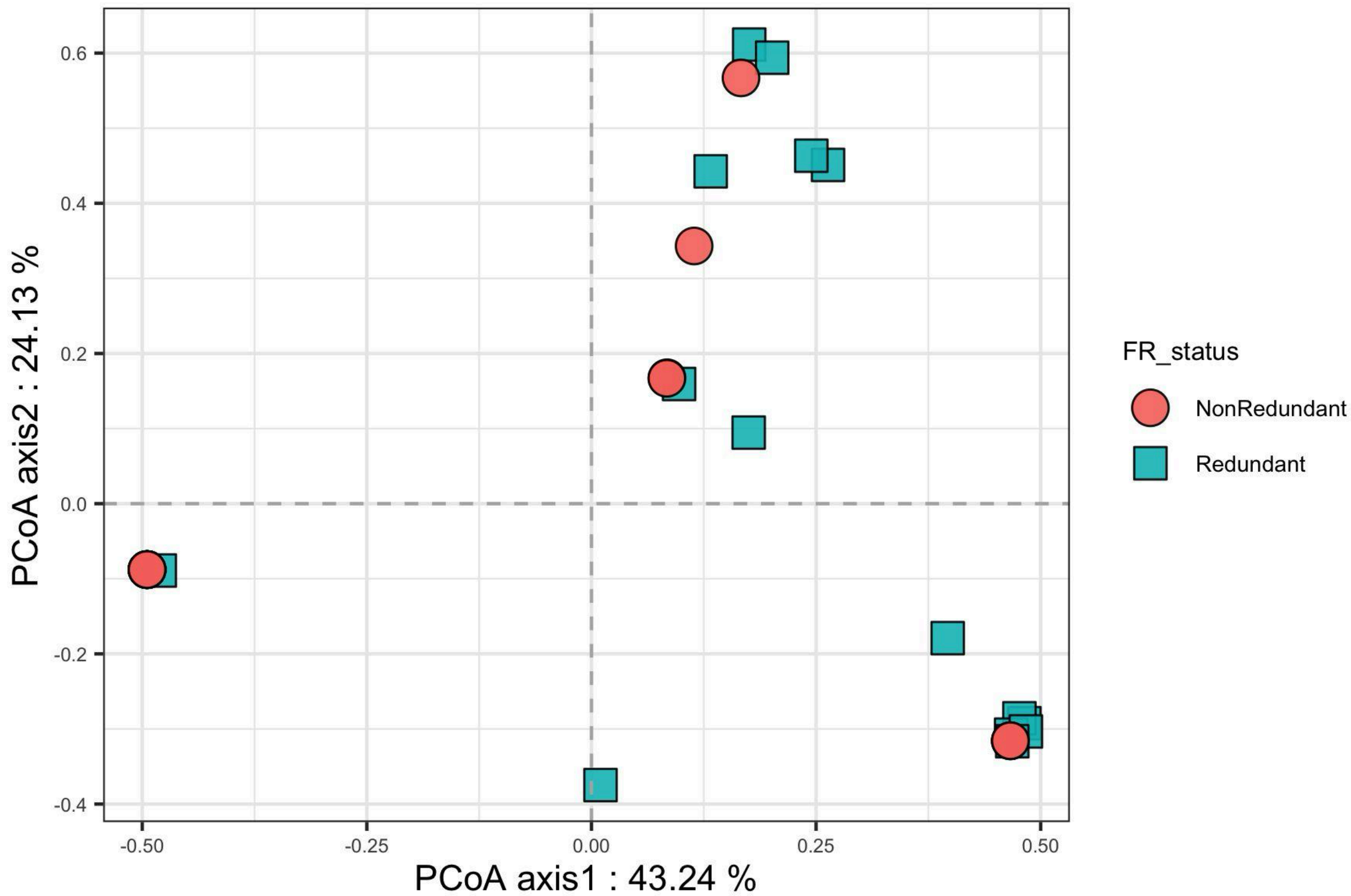

Fig S4. Principal coordinate analysis (PCoA) based on Bray–Curtis dissimilarity showing the similarity between non-redundant (red circles) and redundant (blue squares) resistome communities. Group differences were assessed using PERMANOVA (pseudo-$F = 2.72$, $R^2 = 0.081$, $P = 0.042$; 999 permutations). Homogeneity of dispersion was evaluated using betadisper ($P = 0.026$).

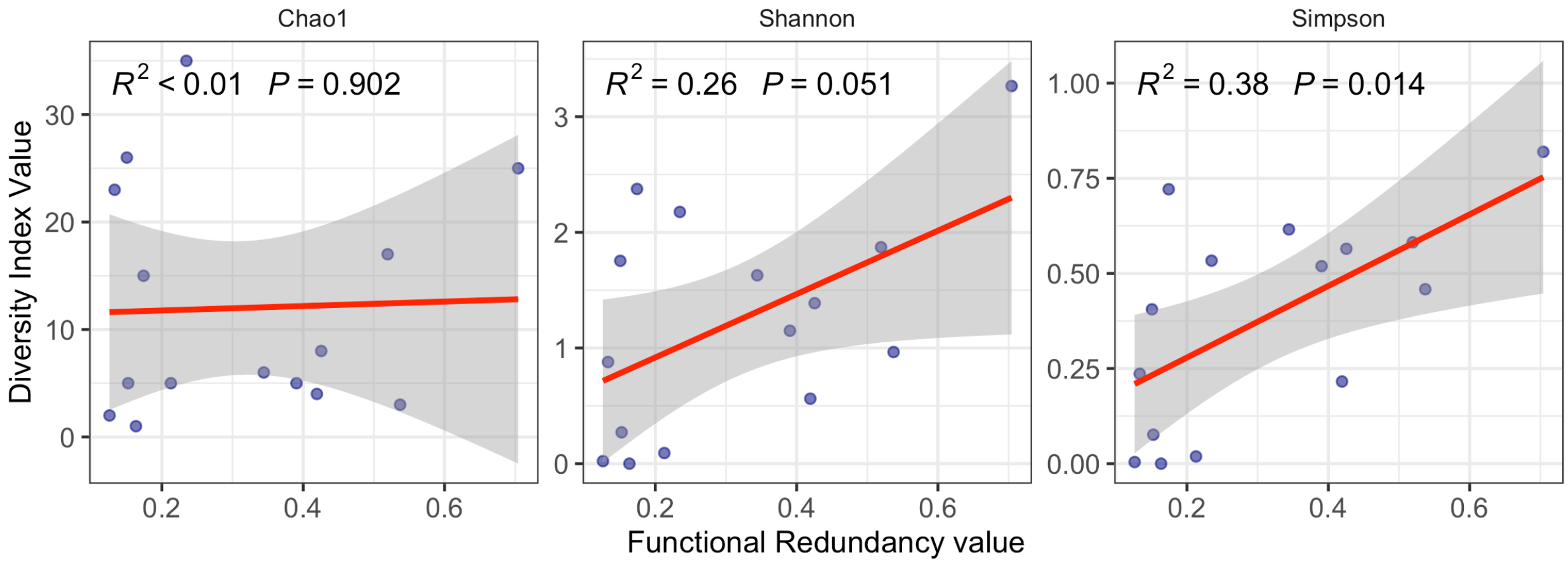

Fig S5. The linear regression model showing the relationship between functional redundancy value and alpha diversities (left: Chao1, middle: Shannon, right: Simpson).

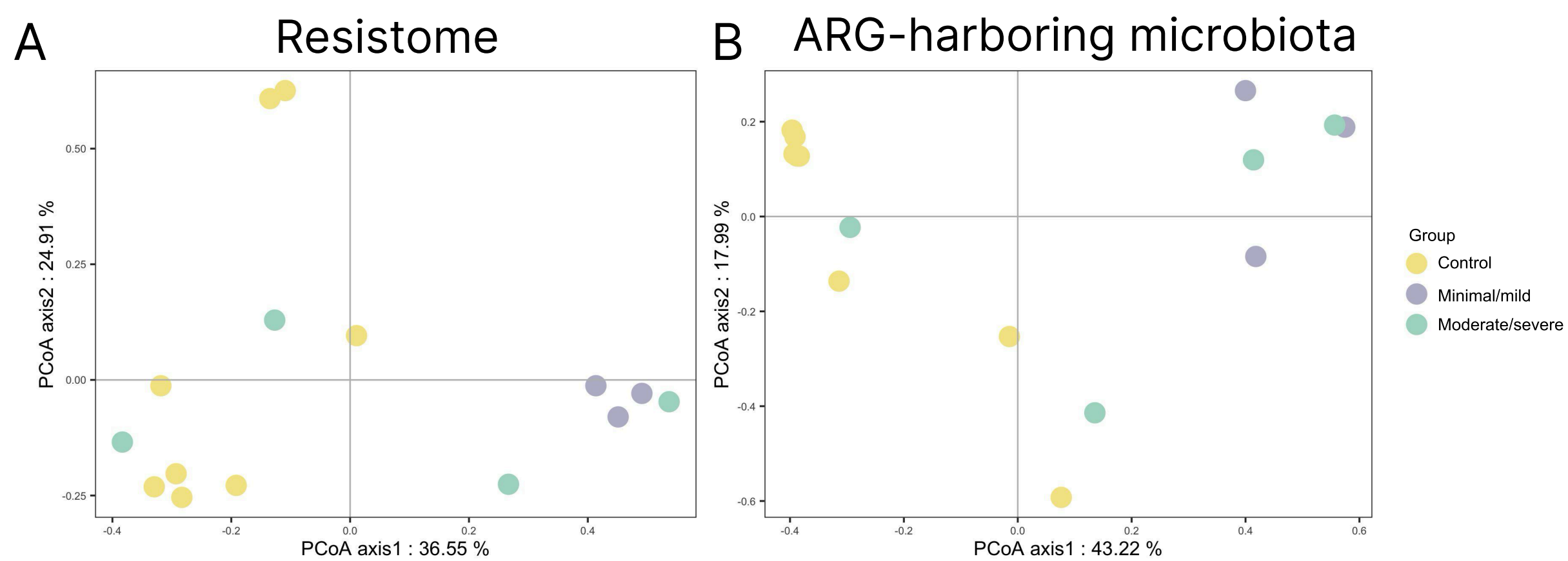

Fig S6. Principal coordinate analysis (PCoA) showing distinct compositional patterns of the resistome (A) and ARG-harboring microbiome (B) among the three clinical groups in the redundant community.

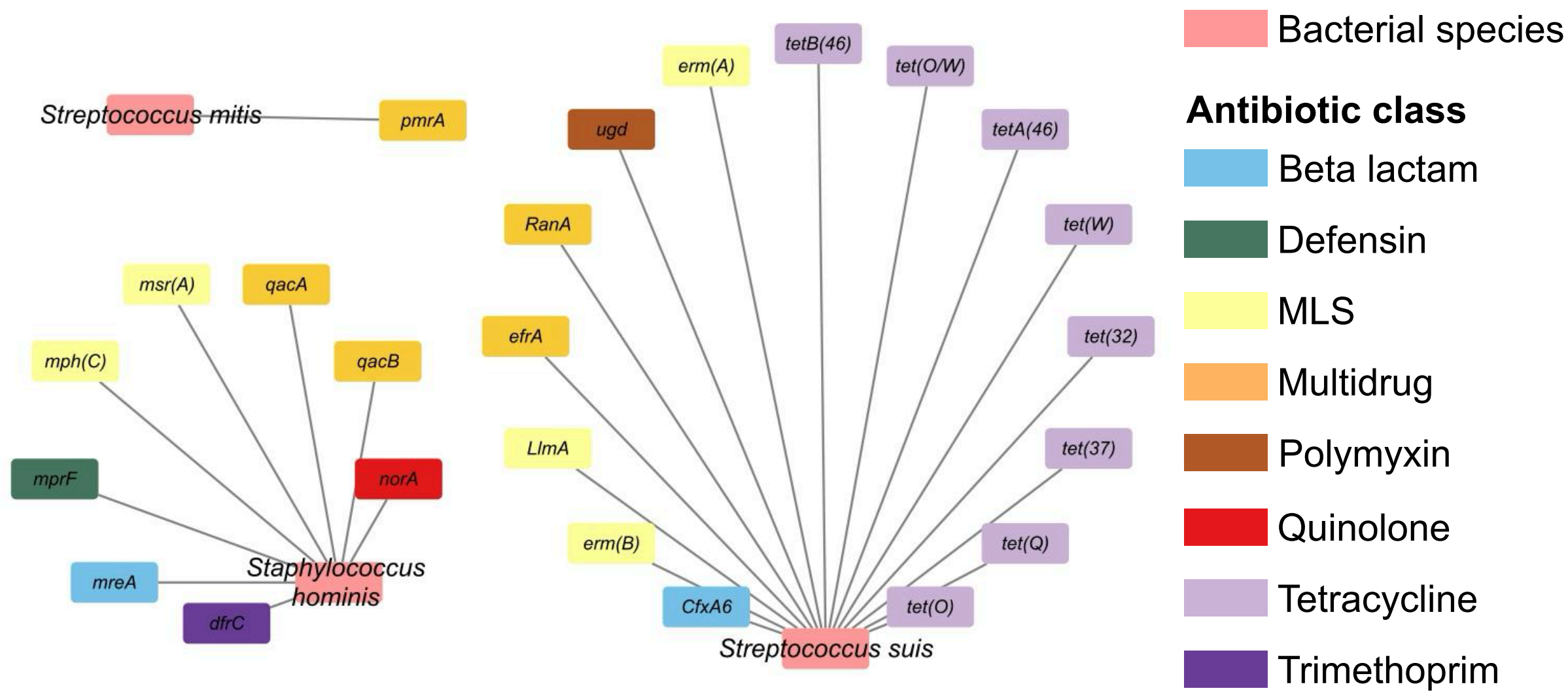

Fig S7. The co-occurrence network between bacterial species and ARGs in healthy controls. The color of each ARG refers to the class of antibiotic to which the gene is predicted to confer resistance.

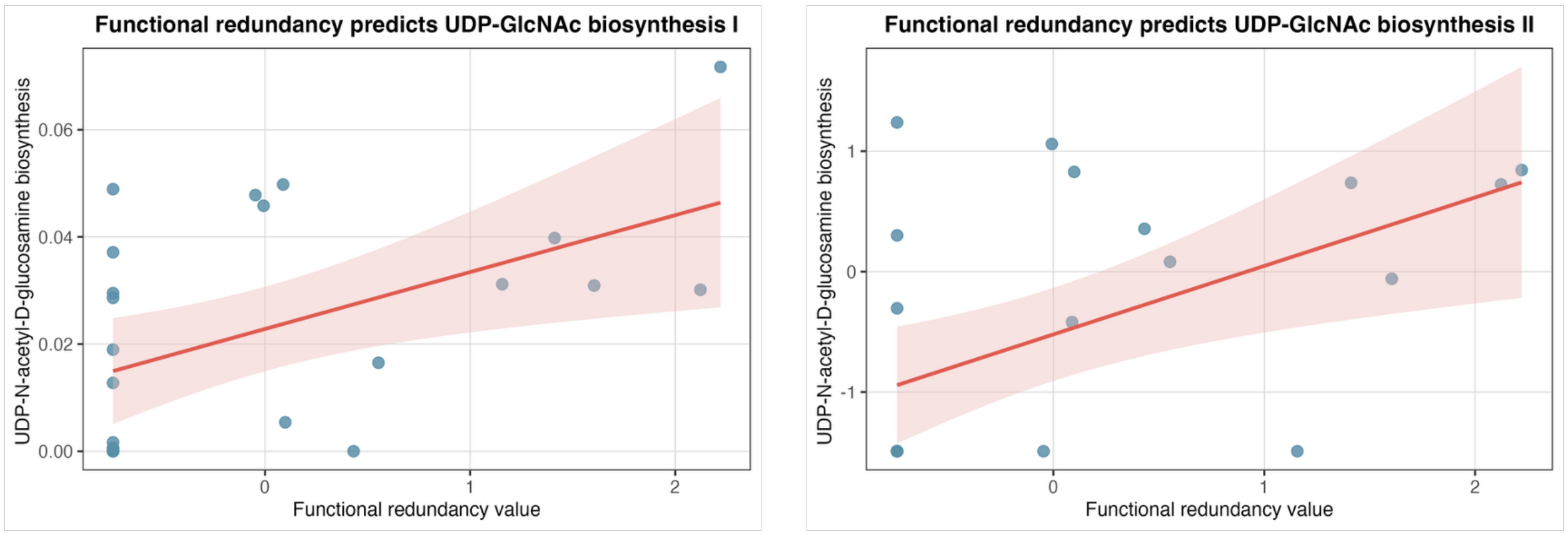

Fig S8. Functional redundancy predicts UDP-N-acetyl-D-glucosamine biosynthesis pathways. Scatter plots show the relationship between functional redundancy values and the predicted abundance of UDP-N-acetyl-D-glucosamine biosynthesis I (left panel) and UDP-N-acetyl-D-glucosamine biosynthesis II (right panel). Each point represents an individual sample. Solid red lines indicate fitted linear regression models, with shaded areas representing 95% confidence intervals.

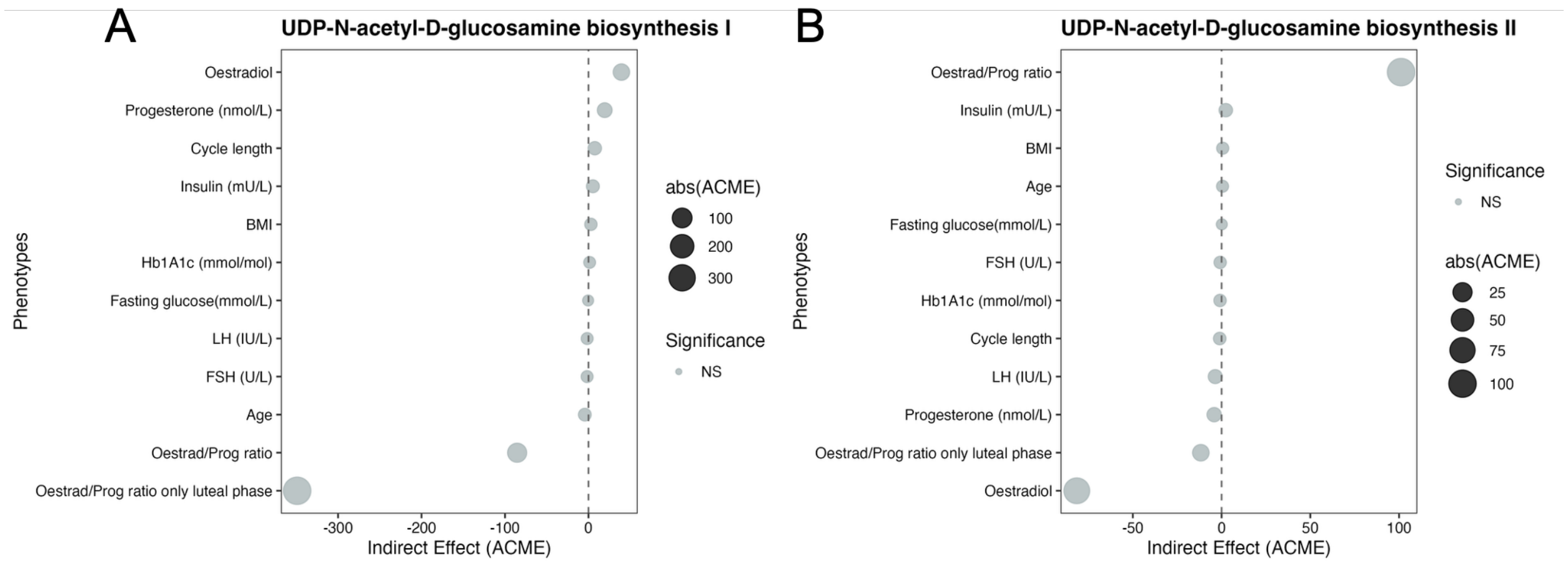

Fig S9. Mediation analysis of host phenotypes through UDP-N-acetyl-D-glucosamine biosynthesis pathways.

Dot plots show the estimated average causal mediation effects (ACME) of host clinical and endocrine phenotypes mediated through UDP-N-acetyl-D-glucosamine biosynthesis I (A) and UDP-N-acetyl-D-glucosamine biosynthesis II (B). Each point represents the ACME estimate for a given phenotype, with point size proportional to the absolute value of the ACME. The dashed vertical line indicates zero indirect effect. Red points denote statistically significant mediation effects, whereas grey points indicate non-significant effects.
